# Assessing the Role of Collective Efficacy Beliefs During Participative Occupational Health Interventions

**DOI:** 10.3389/fpubh.2021.797838

**Published:** 2021-11-25

**Authors:** Marco Kuchenbaur, Richard Peter

**Affiliations:** Institute of the History, Philosophy and Ethics of Medicine, Ulm University, Ulm, Germany

**Keywords:** participative intervention, collective efficacy beliefs, process evaluation, occupational health, questionnaire

## Abstract

**Background:** For group-based participatory interventions in the context of occupational health, no questionnaires exist to assess the participants' active engagement in the interventions. On the basis of the construct of collective efficacy beliefs, this study has developed a questionnaire with which the group-related efficacy beliefs can be assessed as a precondition for participants actively engaging in participative interventions.

**Methods:** Participants were drawn from a two-arm cluster-randomized intervention study to fill out the questionnaire. A Factor analysis and an initial psychometric calibration were performed. In a second step, the group-related properties of the questionnaire were validated using a Multilevel analysis.

**Results:** The factorial structure of the questionnaire is consistent with the theory of efficacy beliefs according to A. Bandura. Furthermore, the collective efficacy expectations of the interventions' participants are lowered in the absence of appreciation and support in the psychosocial environment of the worksite.

**Conclusions:** Assessing participant's quality of interventional activity in participatory interventions by collective efficacy can be valuable in understanding the amount of interventional activity. In addition, it is recommended to consider the influence of the worksite's psychosocial environment on collective efficacy beliefs when implementing participatory interventions.

**Clinical Trial Registration:** Registration trial DRKS00021138 on the German Registry of Clinical Studies (DRKS), retrospectively registered on 25 March, 2020.

## Introduction

An increased interest in process evaluations has emerged in recent years, particularly in complex occupational health intervention studies. This is due to the fact that process evaluations can be highly valuable for understanding how discrepancies between the expected and observed outcomes can be related to context and process of implementation of interventions (1). Whether or not a complex intervention is implemented effectively depends on the quality of intervention activities participants are committed to (2). In complex interventions such as participatory occupational health interventions, the quality of intervention activities is particularly important, as the intervention's success depends on the active engagement of the participants (3).

Human agency does not just begin with cognition over potential actions, but already with the expectancy of mastery of this certain action (4). The concept of so-called efficacy beliefs is a precursor of action and is influenced by individual and group-related factors that facilitate or potentially impede behavior. In case of strong efficacy beliefs, a person or group is convinced that his/her/their behavior will lead to a desired outcome. Efficacy beliefs can be assessed on an individual as well as on a collective level (5). Accordingly, as in participatory occupational health interventions, goal achievement oftentimes require the cooperation of all participants over a longer period of time, an interdependent effort by all participants is necessary for intervention activities (6). The benefit of assessing efficacy expectations in comparison to concrete behavior to indicate active engagement, is that efficacy expectations can be an indicator of the willingness to tackle difficult situations and, above all, to maintain their mastery (4).

In the context of occupational health interventions, Nielsen et al. (7) showed that employees' appraisals of the intervention influenced the relationship between participation and intervention outcomes. There is also empirical evidence that shared participation influences the belief in a so-called occupational self-efficacy (8). Research in the field of implementing standardized workplace interventions indicate that the intervention's activity of participants varies with the belief in individual mastery, i.e., self-efficacy (9). Furthermore, a general and comprehensive attempt to theoretically underpin implementation processes has already been made by May (10). He emphasizes the relevance of social cognitive psychology (i.e., efficacy beliefs) for understanding shared commitment to interventional activity. In addition, efficacy beliefs are part of the “Consolidated Framework for Implementation Research,” a guideline for evaluating complex interventions (11).

Occupational health interventions represent a special category of interventions, as they require a shared commitment due to interdependence structures within organizations (12). The need for shared commitment becomes even clearer when looking at intervention programs that require high engagement from the participants themselves. In participatory interventions based on “Health circles” (3), participants are expected to be involved in the development as well as the implementation of intervention measures. Intervention measures originate from the suggestions of employees themselves, the process of implementation highly depends on participation, i.e., how engaged participants are in the intervention processes. To assess shared commitment as a precondition of collective action, a content-adequate questionnaire should be able to reflect the interactive, coordinative and synergistic dynamics of the task demands (6).

The literature for related questionnaires reveals that many of them are based on the conceptual basis of “Organizational readiness for change” (13), a related construct of efficacy beliefs (see Table 1).

**Table 1:** Content adequacy of existing questionnaires for assessing engagement in participative intervention.

**Questionnaire**	**Type of intervention**	**Participants**	**Attitude object**	**Conceptual basis**	**Adequacy**
Jung et al. (14)^*^	Not specified	Participants from various branches	Health promotion capacity	Health promotion willingness	(+) Level of analysis: Organizations
					(–) Reflection of intervention generation process
Mueller et al. (15)^*^	Non-participative	Participants from various branches	Organizational change	Organizational readiness for change	(–) Type of intervention
					(–) Reflection of intervention generation process
					(+) Level of analysis: Organizations and Individuals
Randall et al. (16)	non-participative	Healthcare workers	Organizational-level stress	Appraisals of	(–) Type of intervention
			management interventions	intervention process	(–) Reflection of intervention generation process
					(+) Consideration of leadership support
Shea et al. (17)	not specified	Students	Organizational change	Organizational	(–) Reflection of intervention generation process
				readiness for change	(–) Participants
					(+) Level of analysis: Organizations and Individuals

* *Listed in Review of Kien et al., “–” not a content-adequate aspect, “+” content-adequate aspect*.

Some questionnaires reflected the shared commitment toward implementation but not toward the beforehand necessary collective generation of intervention measures. The types of interventions addressed by the questionnaires are predominantly standardized programs, rather than interventions developed in a group based participatory process. A review of instruments and outcomes of implementing psychosocial interventions in worksites shows that there are only a few measures available in the context of occupational health interventions (18). All questionnaires found for the purpose of this paper (8, 14–17) are not sufficiently content-adequate for assessing shared commitment in participative, occupational health interventions within group settings like “Health circles” (see Table 1). Although the need for efficacy beliefs is taken into account by some questionnaires, they do not consider the dynamics mentioned by Bandura that characterize the shared cognition of a group that develops and implements interventions together. None of the found questionnaires for process evaluation addresses the role of collective efficacy beliefs to assess participants' shared beliefs of mastering the development and implementation of occupational health interventions exactly.

This paper presents a pilot study of the development and exploratory validation of a questionnaire which assesses participant's overall quality of interventional activity within a group-based, participative intervention program. We assume that collective efficacy expectation moderates the quality of the intervention activity of the participants (19–21). Furthermore, we assume that collective efficacy beliefs are influenced by the psychosocial environment of the workplace. In this study we define collective efficacy beliefs in group-based, participative interventions as the collectively shared beliefs in mastery of developing and implementing intervention measures for occupational health.

## Methods

The study was conducted within the context of the main study, a prospective, two-arm, cluster-randomized intervention study with healthcare workers in seven general and three specialized hospitals, and an elderly care center in Germany, whose wards constitute the clusters (22). At baseline, the intervention arm comprised 22 clusters (*N* = 174 workers). The methodical procedure of this paper is a two-stage process. First, a questionnaire was developed to assess the efficacy beliefs of the interventions participants. In a second step, this questionnaire outcome was validated using a multilevel analysis (MLA) on the basis of validated instruments: the “Effort-Reward-Imbalance questionnaire” and “Copenhagen Psychosocial questionnaire” from the main study's baseline survey. All analysis was conducted with the statistical environment R.

### Conceptual Framework

Defining collective efficacy beliefs as a function of interventional activity entails the following: the perceived difficulty of intervention activity, the strength in terms of duration of the intervention and context-relevant factors of support. We assume that the psychosocial environment of the worksite can be characterized as the context-relevant source of impediments and facilitators of efficacy beliefs (23). In general, the psychosocial environment of the worksite is determined by control and support (24). The model of “Effort-Reward-Imbalance” describes this environment as characterized by interpersonal relationships that are based on mutual cooperative investments, i.e., efforts and the expectancy of an equalization of these efforts, i.e., rewards. Positive self-experiences on the basis of a balance between efforts and rewards can be conducive to strong efficacy beliefs and therefore activity change (25). Likewise, support structures at the interpersonal level can also be conducive to activity initiation: Although Albert Bandura does not directly suggest the influence of leadership support on collective efficacy beliefs at worksite, some studies do show this relationship. Chen and Bliese (26) for example showed that leadership climate is a predictor of collective efficacy in particular. Furthermore, the role of supervisor-employee relationship has been identified as influential in determining employees' willingness to participate in health promotion programs (27). Employees often have no direct influence on social conditions and institutional practices in settings like the workplace. Leadership support represent a form of proxy control for collective agency in this context (6) and is therefore given special consideration.

**Figure 1:**
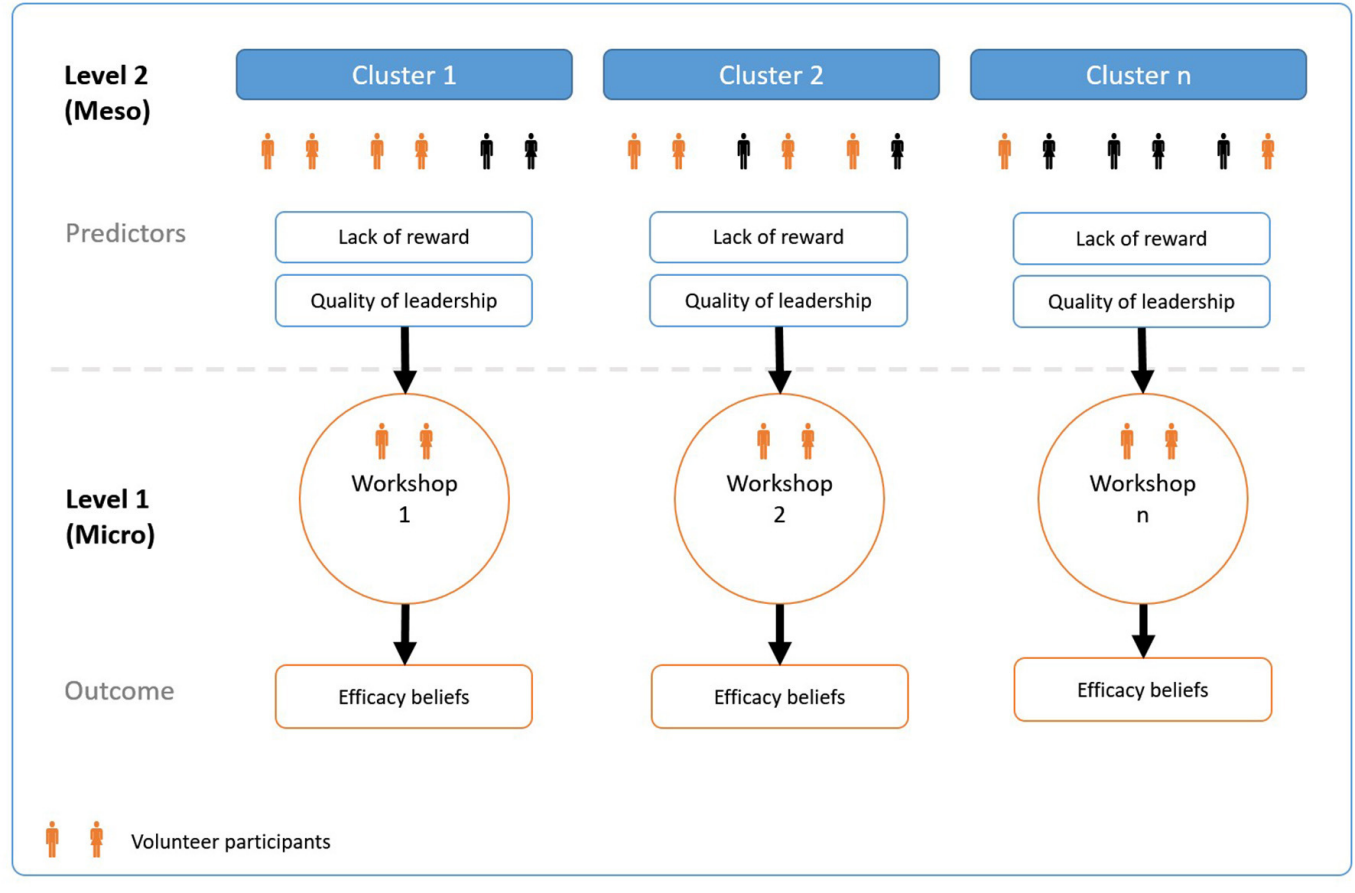
Classification graph for the multilevel context of participative intervention groups.

By assessing participants' collective efficacy beliefs in the context of participative interventions at work, we can draw conclusions about the observed variation to theoretical assumptions about the questionnaire. By using the nested data structure of the pilot study, it is possible to check the questionnaire's accuracy in reflecting the collective efficacy beliefs of the participants in the intervention. Based on the theoretical considerations above, differences in the participants' efficacy beliefs should vary with the quality of the psychosocial environment at worksite. Figure 1 illustrates the nested data structure and relationship between the two levels in the pilot study. The participatory interventions are organized in groups at level 1 and are derived from the organizational units of level 2, e.g., wards or departments. At this second level factors of control and support, as mentioned above, frame the psychosocial environment of interventions participants.

### Participants and Procedure

The participative intervention consisted of interviews and workshops, in which employees participated to develop measures of organizational change, reducing the physical and psychosocial burden. Participants (*N* = 125) were drawn from the intervention arm (*N* = 174) of the cluster-randomized intervention study (see Table 2). They voluntarily engaged in the workshops for developing intervention measures. All workshops (*N* = 24) were assigned to the corresponding clusters (*n*_*j*_ = 22), i.e., organizational units in which the interventions are implemented. Sometimes more than one workshop was conducted per cluster. At the end of the workshop, participants were asked to fill out the questionnaire. Demographic data was collected by assessing age and gender.

**Table 2:** Participants characteristics (*N* = 125).

**Variable**		**Frequency**	**Percent**	**Missing**	**Percent**	**Mean**	**SD**
Age		114	91.2	11	8.8	43.4	11.8
Sex	Female	92	73.6	8	6.4		
	Male	25	20.0				

### Item Generation

For the construction of efficacy scales, an accurate analysis of the domain of functioning (developing and implementing interventions) is necessary (28). Detailed knowledge of the activities within the domain is useful to define factors over which people can exercise control in participative, occupational health interventions. For this purpose, a literature review was conducted in order to first characterize the domain of interventional activities by participants and second to gradate these task demands of the domain against facilitators and impediments of successful performance. Based on existing reviews (29–32), different clusters of the domain of functioning were identified. We considered all factors which are in potential control of those, engaging in a participative intervention. Out of 173 factors identified in the reviews, 63 factors were regarded as relevant for participative interventional activities (Table 3).

**Table 3:** Functional aspects of developing and implementing interventions.

**Dimension**	**References**	**Item**
Challenge of implementation	(29–31)	“I feel that our ward is capable to implement the interventions successfully”
Coherence	(29–31)	“The workshop's goal was present permanently”
Enjoyment and motivation	(29–31)	“I am looking forward to the changes in our organizations the interventions will bring”
Influence	(30, 31)	“All participants had the opportunity to voice their concerns”
Interaction	(29–31)	“Our ward actively engaged in the workshop”
Perception of the program	(29–31)	“Our team was distant toward the workshop (–)”
Support	(29–31)	“We'll receive support from our supervisor for implementing our interventions”

Second, items were developed on the basis of this summary and reviewed by a team of independent scientists (MK, RP). All items were scored on a five-point Likert scale from “not at all” to “to a very high extent.” In the end 26 items resulted and were clustered within seven content domains, reflecting the domain of functioning of developing and implementing participative interventions. Third, items were reviewed by a group of participants (*N* = 8) as part of a comprehension probing (33). Volunteers among workshops' participants were asked to discuss issues of understanding, practicability and purpose of the questionnaire on the basis of a semi-structured interview. Participants were interviewed after they filled out the questionnaire, discussions were transcribed verbatim.

### Exploratory Factor Analysis and Psychometric Properties

Raw data of item responses showed only few missing values per item. Missing values were excluded case-wise. Descriptive data analysis for item distributions, mean and standard deviation, minimum, maximum, and skewness was conducted for analyzing ceiling and floor effects. As ceiling effects were detected, it was decided to use a factor extraction- and estimation method where normal distribution is not a precondition. Furthermore, because anchors of the scales are not necessarily equidistant, it was decided to treat items at an ordinal level of analysis. In cases of non-normal distribution of ordinal data, a suggested method of estimation is the method of unweighted least squares (ULS). ULS yields to more accurate estimations of factor loadings than maximum likelihood in this case (34). Factorability was assessed by calculating individual and overall Kaiser–Meyer–Olkin-Measure (KMO), items with an individual KMO below .70 were excluded (35). The factors were extracted out of a polychoric correlation matrix. For determining the number of factors to be extracted, a parallel analysis (36) was conducted on the basis of this matrix. Scale appropriateness was inspected with classical test theory. The scale score of the two subscales was obtained by calculating the scale mean score. Internal consistency was assessed by calculating Cronbach's alpha.

### Multilevel Analysis for Construct Validation

By using MLA, we aim to reveal the multilevel character of our questionnaire by decomposing its variance components that are determined by factors of the higher-level social structure of the worksite and of individual factors of the participants.

#### Composition Model

As the questionnaire is based on the underlying assumption about a collective level of efficacy beliefs, we consider them as an attribute of the workshop group, shared by the respective members (5). Therefore, the composition model postulated here assumes an additive approach (37) to describe the interaction between the level of workshops (level 1) and its comprising clusters (level 2), representing worksite factors of the nested system. For construct validation, we considered level 2 predictors that are both theoretically plausible and, in addition, allow reliable aggregation on the basis of the intraclass correlation coefficient ICC (2). As a rule of thumb, all aggregated values above .50 were considered (38). Based on the criterion of reliable aggregation, the following subscales from the instruments used in the baseline survey were used as level 2 predictors.

#### Effort-Reward-Imbalance

A predictor of beneficial efficacy beliefs in the context of a positive psychosocial work environment is the Effort-Reward-Imbalance (25). We considered “*Lack of reward*” (α = 0.79) as a plausible predictor since it takes into account the components esteem and job promotion in relation to supervisors and colleagues (39). Items were measured on a four-point scale.

#### Copenhagen Psychosocial Questionnaire

Another indicator to assess the characteristic of the participants' psychosocial environment is the “*Lack in quality of leadership*” (α = 0.92) at cluster level. Quality of Leadership is part of the Copenhagen Psychosocial Questionnaire (40). Research has shown that supervisor behavior can influence the perception of collective efficacy (41). Items were measured on a four-point scale.

The corresponding values for MLA are computed by aggregation, using the arithmetic mean of individual level data that represent the cluster at the baseline survey of the main study. The reliability of the aggregation was checked by calculating the intraclass correlation coefficient ICC (2, 42) and the correlation between individual and aggregated values.

#### Data Analysis Strategy

We used Multilevel analysis (MLA) with Maximum Likelihood estimation to predict level 1 efficacy beliefs by level 2 aggregated scale means with a random intercept model. Since there is no level 1 predictor, the level 2 predictor can only be added to the level 2 intercept equation. First of all, we formulated an unconditional model that decomposes the variance in efficacy into individual variation between workshop participants and group variation between the workshops.


$$\begin{array}{l} \mathrm{Level 1:}\;Y_{ij}=\beta_{0j}\;+\;r_{ij}\\ \mathrm{Level 2:}\;\beta_{0j}=\gamma_{00}\;+\;u_{ij}\\ \;Y_{ij}=\gamma_{00}\;+\;u_{ij}\;+\;r_{ij} \end{array}$$

The workshop-related, individual efficacy expectations *Y*_*ij*_ are modeled as a function of the grand mean of the inter-workshop efficacy expectations and a residual term. Since the rules of composition suggest a random intercept model due to the lack of level 1 predictors, the level 2 predictors on cluster-level can be added to the intercept equation only. Accordingly, the equation for level 2 cluster-mean centered predictors indicate the following structure:


$$\begin{array}{l} \text{Level 2:}\;\beta_{0j}=\gamma_{00}\;+\;\gamma_{01}ERI_{reward\text{\_}mean}\\ \;+\;\gamma_{02}Quality\;of\;Leadership_{mean}\;+u_{ij}\;+r_{ij} \end{array}$$

Based on existing research, we expect that efficacy expectations will be influenced by cluster-related characteristics such as assessed by shared perceptions of a “Lack of reward” γ_01_in the clusters. Substituting the equation above, the following can be derived for level 1:


$$\begin{array}{l} \text{Level 1:}\;Y_{ij}=\gamma_{00}\;+\;\gamma_{01}ERI_{reward_{mean}}\\ \;+\;\gamma_{02}Quality\;of\;Leadership_{mean}\;+\;u_{ij}+\;r_{ij} \end{array}$$

## Results

### Exploratory Factor Analysis and Scale Development

Of the 145 participants in the workshops we received 140 questionnaires (response rate = 0.97). A total of 125 participants in 24 workshops, represented by 22 clusters were used for exploratory factor analysis. Of these, 25 (18.6%) were male, 92 (75.0%) female. 6.4% did not indicate their gender. Fifteen cases were excluded due to missing values. Except for one item, all others were negatively skewed (see Table 4).

**Table 4:** Item descriptive statistics and scale reliability analysis (*N* = 125).

	**Mean**	**SD**	**Skew**	**Kurtosis**	**Item**	**Item**	**α if**
					**difficulty**	**discrimination**	**deleted**
**Workshop-related efficacy expectation^a^**							
Our team was distant toward the workshop (–)	4.22	0.99	−1.47	2.05	0.84	0.62	0.74
The participants didn't made much proposals during the workshop (–)	4.46	0.92	−2.11	4.44	0.89	0.58	0.75
All participants had the opportunity to voice their concerns	4.76	0.51	−2.45	7.24	0.95	0.47	0.77
I was able to bring my demands to the discussion in the workshop	4.53	0.56	−0.94	1.5	0.91	0.54	0.76
I expect my work situation worsening throughout the intervention (–)	4.27	0.96	−1.62	2.5	0.85	0.46	0.76
The interventions reflect my personal demands	4.26	0.62	−0.64	1.48	0.85	0.48	0.76
All participants supported the decisions made	4.54	0.56	−0.7	−0.55	0.91	0.43	0.77
Our ward actively engaged in the workshop	4.43	0.81	−2.17	6.46	0.89	0.32	0.78
In the Workshop there were discussions about useful interventions for my team	4.45	0.64	−1.67	6.46	0.89	0.38	0.77
I am sceptical toward the interventions (–)	3.49	1.07	−0.48	−0.51	0.7	0.33	0.79
The workshop's goal was present all the time	4.46	0.67	−1.33	2.55	0.89	0.37	0.77
**Prospective outcome expectations^b^**							
The presented interventions can be implemented in future	4.38	0.58	−1.29	7.51	0.88	0.46	0.72
I regard the interventions as useful for my ward	3.98	0.77	−0.94	1.23	0.8	0.54	0.7
I expect that the interventions will reduce my problems at work	3.99	0.77	−0.86	1.02	0.8	0.49	0.71
I feel that our ward is capable to implement the interventions successfully	4.02	0.63	−0.97	2.76	0.8	0.41	0.72
Our ward is able to cope potential challenges of the implementation	3.82	0.81	−1.05	1.37	0.76	0.41	0.72
We'll receive support from our supervisor for implementing our interventions	3.99	0.9	−0.85	0.17	0.8	0.38	0.73
I am convinced that we in our department are giving each other sufficient support for the implementation	4.05	0.71	−1.05	2.18	0.81	0.4	0.72
I am looking forward to the changes in our organizations the interventions will bring	3.85	0.81	−0.81	0.97	0.77	0.44	0.72
I am positively affected by the interventions within my workspace	4.16	0.71	−0.92	1.62	0.83	0.3	0.74

a *Mean inter-item-correlation = 0.267. Cronbach's α = 0.783*,

b *Mean inter-item-correlation = 0.249. Cronbach's α = 0.74; SD, Standard deviation*.

#### Comprehension Probing

The participants rated the items as comprehensible and were able to anticipate the purpose of the questionnaire. The understanding of one item was classified as inconsistent because the wording was contradictory. For this reason, it was excluded from further analysis and development.

#### Factor Analysis and Reliability

An initial, unweighted least square estimation of the factors (ULS) on the basis of a polychoric correlation matrix was conducted on the 26 items with orthogonal rotation (varimax). The overall KMO verified a “middling” sampling adequacy for the analysis (KMO = 0.73), five items were excluded due to mediocre individual KMO. Bartlett's test of sphericity, $\chi_{\left(25\right)}^{2}$ = 287.5214, *p* < 0.001, indicated that correlations between items were sufficiently large. Parallel Analysis (36) suggested a two-factor-solution, concerning eigenvalues over Kaiser's criterion of 1. This two factors were retained in the final analysis. Table 5 shows the factor loadings after rotation. Factor loadings of 0.40 and above were considered as salient for further scale development (43). Of the 26 items, 20 loaded saliently on one of the two factors. Items that cluster on the same factors suggest that factor 1 represents efficacy expectations toward the intervention in workshops, whereas factor 2 items represent prospective outcome expectations of the intervention and its implementation. This factor structure is consistent with the dichotomous scheme of efficacy beliefs, which distinguishes between efficacy expectations and outcome expectations (4). Both factors showed reasonable standardized internal consistency, factor 1 (α = 0.78) and factor 2 (α = 0.75), and acceptable values for the mean-inter-item-correlation within the range (0.15–0.50) (44), see Table 4.

**Table 5:** Two-factor solution for the 20 Likert-scaled items (*N* = 125) after varimax rotation.

	**Factor 1**	**Factor 2**	**Communality**	**Uniqueness**
**Workshop-related efficacy expectation**				
Our team was distant toward the workshop (–)	0.736		0.552	0.448
The participants didn't made much proposals during the workshop (–)	0.726		0.544	0.456
All participants had the opportunity to voice their concerns	0.682		0.510	0.490
I was able to bring my demands to the discussion in the workshop	0.644		0.505	0.495
I expect my work situation worsening throughout the intervention (–)	0.615		0.390	0.610
The interventions reflect my personal demands	0.527		0.542	0.458
All participants supported the decisions made	0.514		0.491	0.509
Our ward actively engaged in the workshop	0.459		0.278	0.722
In the Workshop there were discussions about useful interventions for my team	0.454		0.393	0.607
I am skeptical toward the interventions (–)	0.448		0.229	0.771
The workshop's goal was present all the time	0.419		0.266	0.734
**Prospective outcome expectation**				
The presented interventions can be implemented in future		0.707	0.551	0.449
I regard the interventions as useful for my ward		0.706	0.550	0.450
I expect that the interventions will reduce my problems at work		0.703	0.503	0.497
I feel that our ward is capable to implement the interventions successfully		0.558	0.338	0.662
Our ward is able to cope potential challenges of the implementation		0.526	0.299	0.701
We'll receive support from our supervisor for implementing our interventions		0.503	0.276	0.724
I am convinced that we in our department are giving each other sufficient support for the implementation		0.497	0.280	0.720
I am looking forward to the changes in our organizations the interventions will bring		0.448	0.277	0.723
I am positively affected by the interventions within my workspace		0.394	0.239	0.761
Total variance after rotation in %	20	19		

#### Model Fit

Since the scree plot was not completely unambiguous, a model with three factors was also calculated. The computed ANOVA test for the two-factor solution showed a better fit compared to the three-factor solution, considering the Bayesian information criterion (BIC). Furthermore, the decision was made in favor of a two-factor solution, since the interpretation of the meaning of factors is better according the fit with Bandura's concept of efficacy beliefs. The two-factor-model accounted for 39% of the total variance. A root mean squared residual (RMSR) of 0.08 was computed, which means that the average of overall residuals was just sufficient to meet the acceptable limit (45).

### Multilevel Analysis

For both “Lack of reward” [*ICC* (2) = 0.54] and “Quality of leadership” [*ICC* (2) = 0.84], a reliable aggregation of the scale means at level 2 was feasible. Age and gender of the participants were considered during modeling process. Both variables had no influence on the presented models. Both scales of the developed questionnaire were tested on the ability for a multilevel analysis, but only scale 1 could provide significant results: Analysis of the unconditional model 1 showed a 35% [*ICC* (1) = 0.35] variation in efficacy expectations due to the grouping in clusters (see Table 6). This confirms the assumption of a hierarchical data structure. A grand mean of workshop-related efficacy expectations (γ_00_ = 4.32, *p* < 0.01) was observed. A stepwise selection algorithm based on the Akaike information criterion (AIC) was performed to determine the best model fit (46). Model 2, which showed the best model fit, accounts for the “Lack of reward” at cluster level. Workshop-related efficacy beliefs (level 1) are reduced by a of “Lack of reward” in the level 2 clusters (γ_01_ = −0.39, *p* = 0.01). Thus, 29% of the variance between the level 1 workshops' efficacy beliefs could be explained by the shared perception of “Lack of reward” at level 2 (cluster).

**Table 6:** Variance component models for efficacy expectations in workshops (*N* = 125).

		**Model 1**			**Model 2**		
**Predictors**	**Parameter**	**Estimates**	**CI**	***p***	**Estimates**	**CI**	***p***
(Intercept)	γ_00_	4.32	4.18–4.45	<0.001	5.6	4.61 to 6.59	<0.001
Lack of reward	γ_01_				−0.39	−0.69 to −0.09	0.01
**Random effects**							
Individual level variance σ^2^		0.13			0.13		
Group level variance τ_00_		0.07 _cluster_			0.05 _cluster_		
ICC		0.35			0.26		
*N*		22 _cluster_			22_cluster_		
Observations		125			125		
Marginal *R*^2^/Conditional *R*^2^		0.000/0.346			0.097/0.336		
**Model fit**							
AIC				136.21			132.37
BIC				144.69			143.69
Log likelihood				−68.3			−62.19

*Model 2: Δχ^2^ = −6.1 (p <0.001); CI, confidence intervals at the 95% level; ICC, Intraclass correlation coefficient*.

### Correlational Analysis

**Figure 2:**
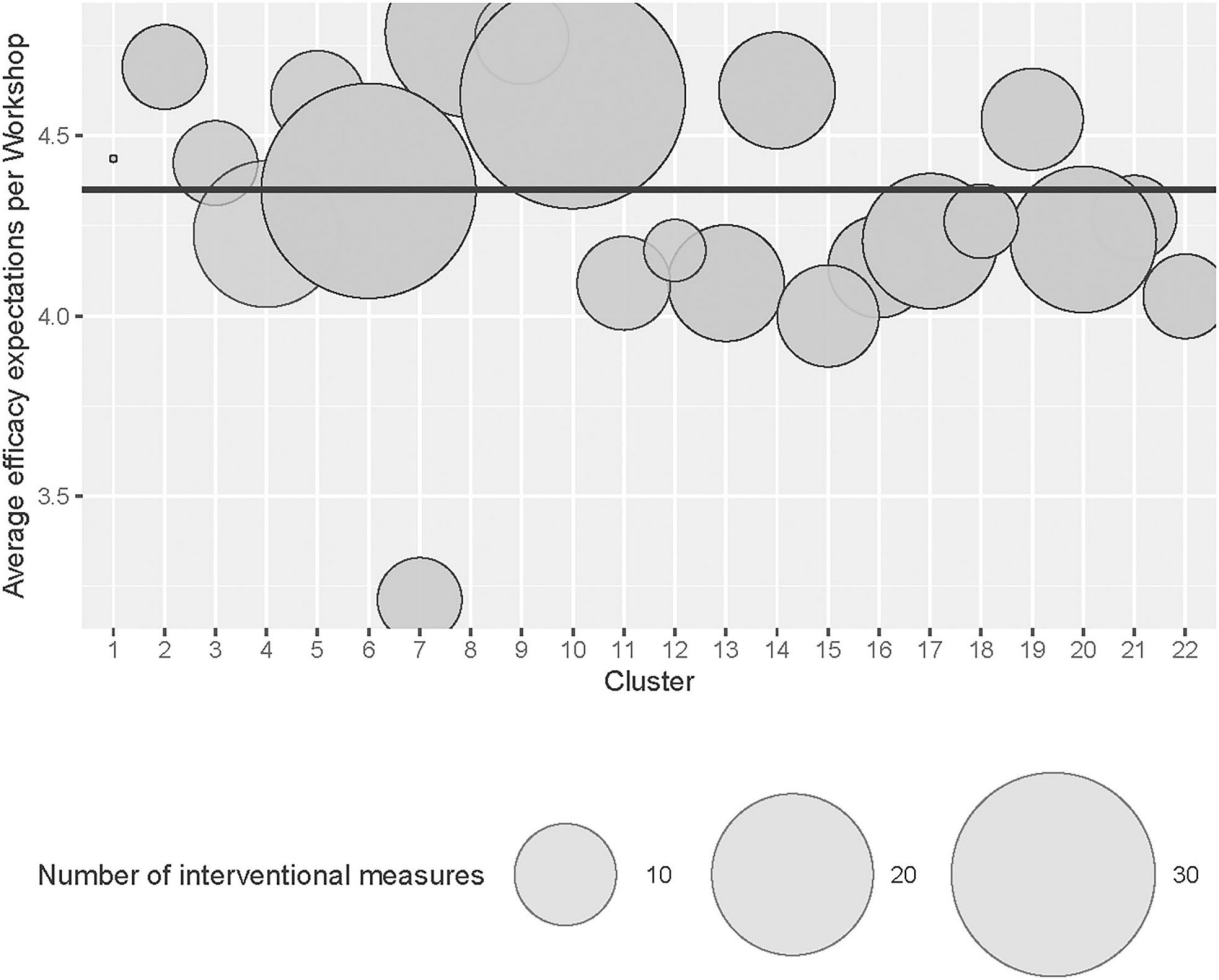
Number of interventional measures across workshop groups (Cluster).

The aim of the questionnaire is to explain the quality of the interventional activity in participative interventions. In order to verify this by correlational analysis, the number of interventional measures that emerged from each workshop was correlated with the aggregated scale score of the 22 workshop groups' efficacy expectation. It is assumed that a higher overall efficacy expectancy of a workshop group is associated with a larger number of interventional measures (see also Figure 2). The analysis showed a moderately strong correlation between the number of interventional measures and workshop-related efficacy expectation (η = 0.57, *p* < 0.001).

## Discussion

This pilot study intended to develop and evaluate a new questionnaire, assessing interventional action of participants of a participative, occupational health intervention. This was achieved by drawing on Bandura's concept of collective efficacy beliefs as a precondition of activity initiation.

The analysis showed that active engagement in participatory interventions is influenced by the psychosocial environment of the participants' worksite. The results of this study suggest that collective efficacy beliefs, as a precursor of interventional action, can map the resources for participant's contribution to the implementation of interventions (10). This is crucial for the understanding of how organizational change in participatory worksite interventions can be realized.

Although previous studies repeatedly emphasize the role of efficacy beliefs for the success of interventions, the actual interactive context in which occupational health interventions are developed has been neglected, since the used intervention measures are predominantly standardized (17, 18). The distinctive challenge of participatory interventions lies in group-based processes of collaboration, support, and potential conflict in the joint identification of intervention measures. These processes are crucial for the collective development of shared efficacy beliefs. To our knowledge, the participatory context of intervention groups in occupational health interventions has not been highlighted by any other study yet.

### Interpretation: Strengths and Mechanisms

The questionnaire's factorial structure is consistent with the dichotomous scheme of efficacy beliefs, which distinguishes between efficacy expectations and outcome expectations (4). According to Bandura's theory, Factor 2 can be interpreted as outcome expectation, since the items are mainly used to assess the future feasibility of the interventions. Factor 1, on the other hand, represents the extent of efficacy expectations of the group reflecting the overall workshop situation with regard to facilitators and impediments.

A particular strength of the study is that the multilevel analysis could confirm the collective-level properties of the questionnaire: The workshop participants' shared perception of the efficacy expectation of the interventions was found to be determined by the psychosocial environment of the clusters, i.e., organizational units, which comprise the workshop. Workshop-related efficacy beliefs are reduced by a “Lack of reward” in the higher-level clusters (γ_01_ = −0.39, *p* < 0.01). Therefore, psychosocial environments that are characterized by the absence of mutual appreciation and respect can reduce a group's members opportunity to experience themselves in a positive way (25), which can affect the efficacy expectations toward an intervention. This finding is also in line with theoretical considerations on the internal and external locus of control (47), according to which expectancy of control is characterized by one's own behavior as well as by situational and structural factors.

Furthermore, the correlation analysis shows that the external criterion of the number of interventional measures is related to the workshop-related efficacy expectation. We can assume that the number of generated interventional measures is influenced by the shared efficacy expectation of the workshop's participants. We were able to show that collective efficacy beliefs can be an indicator for the outcome of participatory interventions.

The results of the study are further supported by the findings of a comprehension probing with semi-standardized questions to eight participants of the workshops. They rated the questionnaire as comprehensible and appropriate to assess the context. The overall objective of the questionnaire was clear to the participants.

### Limitations

Participants were recruited voluntarily. Higher motivated employees presumably agreed to participate in the workshops more often. In quite a few cases, however, the participants' direct supervisors were also present, which may have resulted in some positive skew in responding to the item on supervisor support. Mixed hierarchies within intervention groups may impede the ability to address problems they might have with the supervisor (3). Within comprehension probing, this was also pointed out by one interviewee.

The manifest aggregation of individual data by computing arithmetic means is associated with some problems (48). Latent aggregation methods realized by Multilevel structural equation modeling is a valuable alternative but not applicable in our context due to small number of cases within groups. Nevertheless, to ensure the reliability of the aggregation of the cluster-related individual data, correlations were calculated between both the aggregated scale means at the cluster level and the individual scale means.

Maximum likelihood estimation in the context of multilevel analyses requires adequate sample sizes. The number of groups is more relevant than the number of individuals in this context. In our pilot study though, the number of groups is limited to 22. A simulation study has shown that with a group number of 30, the accuracy of the regression coefficients is achieved. However, the standard error of the level 2 variance is underestimated by about 15% (49). Interpretation of the results should be made with caution.

Caution should be taken too, when interpreting the internal consistency. According to Cortina (50), the coefficient alpha is highly dependent on the number of items. For the interpretation of internal consistency, the average inter-item correlation should therefore also be taken into account. Since the attitude object of scale 1 and 2 represents the intervention measures in general, the value for the inter-item correlation for this broad construct can be regarded as acceptable according to Clark and Watson (44).

For further, more detailed psychometric assessment, future analyses with this questionnaire should include confirmatory tests with larger sample sizes.

### Practical Relevance

Since the questionnaire can be used to assess collectively-shared efficacy beliefs, its use is recommended for participatory interventions at worksite, where aspects of appreciation and support can influence the initiation of intervention activity (3).

## Conclusion

The questionnaire provides a contribution to the question of whether or not an initial interventional activity of the participants of a participatory occupational health intervention has taken place by referring to the construct of collective efficacy beliefs. Moreover, the role of the worksite's psychosocial environment in influencing participants' efficacy expectations was demonstrated. The questionnaire is appropriate for the group-based assessment of efficacy beliefs for participatory interventions in the field of occupational health.

## Data Availability Statement

The raw data supporting the conclusions of this article will be made available by the authors, without undue reservation.

## Ethics Statement

The studies involving human participants were reviewed and approved by Ethikkommission der Universität Ulm. The patients/participants provided their written informed consent to participate in this study.

## Author Contributions

MK prepared and edited the introduction, methods, results, and discussion sections of the manuscript, and conducted the statistical analyses. RP reviewed the introduction, methods, results, and discussion sections of the manuscript. All authors read and approved the final manuscript.

## Funding

The intervention study HALTgeben was funded from 01.02.2019 to 31.01.2022 by the German Federal Innovation Fund under grant agreement 01VSF18006. The funding institution had no influence in the study design, the collection, analysis and interpretation of the data, and the writing of the report.

## Conflict of Interest

The authors declare that the research was conducted in the absence of any commercial or financial relationships that could be construed as a potential conflict of interest.

## Publisher's Note

All claims expressed in this article are solely those of the authors and do not necessarily represent those of their affiliated organizations, or those of the publisher, the editors and the reviewers. Any product that may be evaluated in this article, or claim that may be made by its manufacturer, is not guaranteed or endorsed by the publisher.
