# Corrigendum: Assessing the Role of Collective Efficacy Beliefs During Participative Occupational Health Interventions

**DOI:** 10.3389/fpubh.2021.822431

**Published:** 2021-12-16

**Authors:** Marco Kuchenbaur, Richard Peter

**Affiliations:** Institute of the History, Philosophy and Ethics of Medicine, Ulm University, Ulm, Germany

**Keywords:** participative intervention, collective efficacy beliefs, process evaluation, occupational health, questionnaire

In the original article, there was a mistake in Figure 2 as published, due to the upload of a broken .eps file in the pursuit of a less pixelated rendering of figure 2. The corrected Figure 2 appears below.

**Figure 2:**
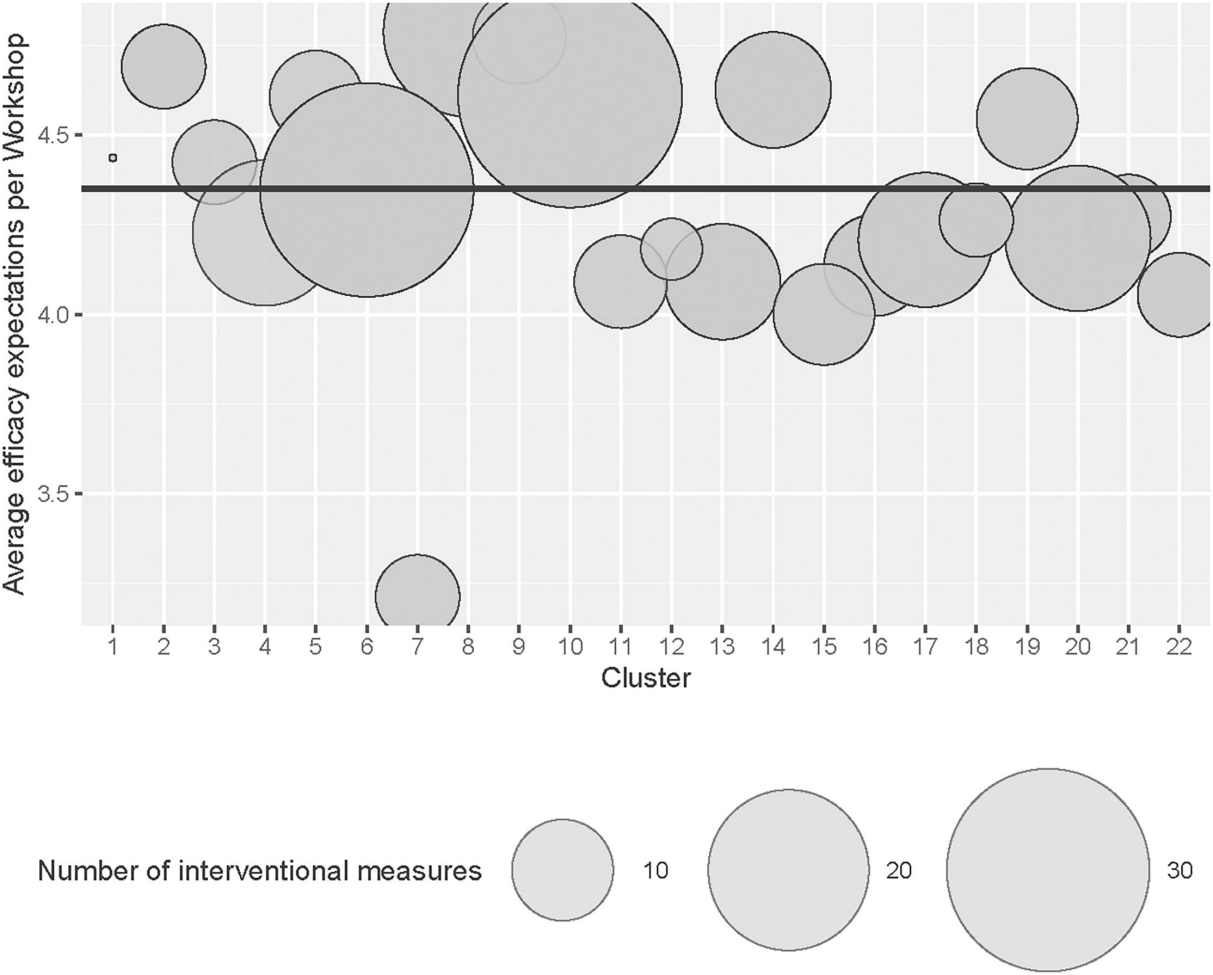
Number of interventional measures across workshop groups (Cluster).

The authors apologize for this error and state that this does not change the scientific conclusions of the article in any way. The original article has been updated.

## Publisher's Note

All claims expressed in this article are solely those of the authors and do not necessarily represent those of their affiliated organizations, or those of the publisher, the editors and the reviewers. Any product that may be evaluated in this article, or claim that may be made by its manufacturer, is not guaranteed or endorsed by the publisher.

